# Epidemiology of alcohol-related unintentional drowning: is post-mortem ethanol production a real challenge?

**DOI:** 10.1186/s40621-018-0169-4

**Published:** 2018-11-01

**Authors:** Tuulia Pajunen, Erkki Vuori, Philippe Lunetta

**Affiliations:** 1 Department of Biomedicine, Pathology and Forensic Medicine, University of Turku, Kiinamyllynkatu 10, 20520 Turku, Finland; 2 Department of Forensic Medicine, University of Helsinki, Kytösuontie 11, 00500 Helsinki, Finland; 3 Department of Forensic Medicine, University of Oulu, 90014 Oulu, Finland

**Keywords:** Unintentional drowning, Alcohol, Post-mortem ethanol production, Epidemiology

## Abstract

**Background:**

Post-mortem (PM) ethanol production may hamper the interpretation of blood alcohol concentration (BAC) in victims of drowning. Different exclusion criteria (e.g. cases with low BAC or with protracted interval between death and toxicological analysis) have been proposed with no factual figures to reduce the potential bias due to PM ethanol production when examining the prevalence rates for alcohol-related drowning. The aim of this study is to verify the extent to which PM alcohol production may affect the accuracy of studies on drowning and alcohol.

**Findings:**

Unintentional fatal drowning cases (*n* = 967) for which a full medico-legal autopsy and toxicological analysis was performed, in Finland, from 2000 to 2013, and relevant variables (demographic data of the victims, month of incident, PM submersion time, blood alcohol concentration, urine alcohol concentration (UAC), vitreous humour alcohol concentration (VAC) were available. Overall, out of 967 unintentional drownings, 623 (64.4%) were positive for alcohol (BAC > 0 mg/dL), 595 (61.5%) had a BAC ≥ 50 mg/dL, and 567 (58.6%) a BAC ≥ 100 mg/dL. Simultaneous measurements, in each victim, of BAC, UAC, and VAC revealed PM ethanol production in only 4 victims (BAC: 25 mg/dL – 48 mg/dL). These false positive cases represented 0.4% of drownings with BAC > 0 mg/dL and 14.3% of drownings with BAC > 0 mg/dL and < 50 mg/dL.

**Conclusions:**

The present study suggests that PM ethanol production has a limited impact on research addressing the prevalence rate for alcohol-related drowning and that the use of too rigorous exclusion criteria, such as those previously recommended, may led to a significant underestimation of actual alcohol-positive drowning cases.

**Electronic supplementary material:**

The online version of this article (10.1186/s40621-018-0169-4) contains supplementary material, which is available to authorized users.

## Background

Post-mortem (PM) endogenous alcohol production may hamper accurate assessment of blood alcohol concentration (BAC) in victims of fatal injuries (Kugelberg and Jones 2007; Appenzeller et al. 2008). A range of micro-organisms can produce ethanol via the process of fermentation, mainly by using glucose as a substrate (Corry 1978). PM ethanol production, if it occurs, usually remains below 70 mg/dL (Gilliland and Bost 1993), although PM production has been reported at even higher than 150 mg/dL (Corry 1978).

PM alcohol production is recurrently mentioned in studies on drowning and alcohol (Lunetta et al. 2004; Ahlm et al. 2013; Pajunen et al. 2017). Accurate blood alcohol assessment is crucial as ethanol has been identified as a major risk factor for drowning especially in adults. Cut-off times for toxicological sampling (Schuman et al. 1978; Hoxie et al. 1988; Wintemute et al. 1990) or exclusion of cases with low BAC (Ahlm et al. 2013; Peden et al. 2017) have been proposed to minimize false-positive alcohol-related cases. However, most studies focus on BAC alone and overlook the potential of concurrent urine and vitreous humour analysis to discriminate ante-mortem (AM) alcohol ingestion from any PM artefact.

The aim of this study was to estimate to what extent PM alcohol production may hamper epidemiological studies on drowning and alcohol by reducing their accuracy.

## Materials and methods

### Design and setting

This retrospective study focuses on drownings that underwent a medico-legal autopsy and toxicological analysis in Finland from 2000 to 2013. Of 2563 fatal drownings, 2015 were identified as unintentional using ICD-10 external cause codes for drowning (V90, V92, W65-W74). Among these, the following cases were excluded: a) date of death not certain or date of body retrieval unspecified (*n* = 736), b) blood samples or both urine- and vitreous-humour samples missing or not analysed for alcohol (*n* = 262) c) deaths at hospital or in an ambulance (*n* = 86). After these exclusions, the study included 967 unintentional drownings for which BAC was available, and urine or vitreous humour or both were also tested for alcohol.

The main assumption was that cases with PM endogenous alcohol production disclose ethanol in blood samples but not in the urine or vitreous humour or in both, as such substrates are less susceptible than blood to PM ethanol production (Kugelberg and Jones 2007).

### Data collection and analysis

The data were collected from two sources (Pajunen et al. 2017). The free-text of the death certificates issued by a medical examiner and supplied by Statistics Finland (permission TK53–277-15) were manually screened for information as to date of death and of body retrieval, site of death (bath, lake, river, pond, sea, well, canal, rapids, ditch, pool, other), and pre-drowning activities (boating, bathing, wading, swimming, land traffic, falling, swimming, diving, other) by a member of our research group. BAC, urine alcohol concentration (UAC), and vitreous humour alcohol concentration (VAC) were extracted from the database of the Laboratory of Forensic Toxicology, Department of Forensic Medicine, University of Helsinki. The relevant data were linked by individual autopsy-case codes.

The same laboratory (Testing Laboratory No. T115) performed PM toxicological analysis for all medico-legal autopsies performed in Finland during the study period. Its analytical methods have been accredited since 1997 by the Centre for Metrology and Accreditation according to SFS-EN 45001 ISO/IEC Guide 25. The blood and urine samples were usually collected at the beginning of the internal examination directly from the femoral vein and urinary bladder; the vitreous humour samples by penetrating the organs with a sterile needle and syringe. The samples were preserved in tubes containing sodium fluoride. BAC, UAC, and VAC were measured by a dual-column head-space gas-chromatography. Four independent measurements are required for the final mean concentration value.

## Findings

Overall, among the 967 unintentional drowning victims, 595 (61.5%) had a BAC ≥ 50 mg/dL and 28 (2.9%) a BAC **>** 0 and < 50 mg/dL (Table 1). In 220 (22.8%) cases, both UAC and VAC were available; in 943 (97.5%), UAC but not VAC; and in 24 (2.5%), VAC but not UAC was available.

**Table 1:** Unintentional drownings, 2000–2013, by PM submersion time and BAC

PM submersion time	No of cases	BAC 0 mg/dL (%)	0 < BAC < 50 mg/dL (%)	BAC ≥ 50 mg/dL (%)
< 1 day	776	296 (38.1)	15 (2.0)	465 (59.9)
1 day	70	26 (37.1)	0 (0)	44 (62.9)
2 to 6 days	69	11 (16.0)	5 (7.2)	53 (76.8)
≥ 7 days	52	11 (21.1)	8 (15.4)	33 (63.5)
Total	967	344 (35.6)	28 (2.9)	595 (61.5)

*BAC* blood alcohol concentration, *PM* post-mortem

The monthly distribution of overall unintentional drownings showed a peak during the summer months. Most victims were recovered from water ≤1 day PM. A statistically significant difference appeared in distribution of death months between groups with BAC 0 mg/dL and BAC ≥ 50 mg/dL (*p* < 0.05, χ2-test; JMP Pro 13.2.1). No statistically significant difference, however, emerged in death months between groups with BAC 0 mg/dL and BAC > 0 and < 50 mg/dL. In addition, a significant difference emerged in distribution of submersion times between groups with BAC 0 mg/dL and BAC > 0 and < 50 mg/dL, and between groups with BAC 0 mg/dL and BAC ≥ 50 mg/dL (*p* < 0.05, χ2-test; JMP Pro 13.2.1) (Tables 1 and 2).

**Table 2:** Unintentional drownings, 2000–2013, by months and BAC

Months of death	No of cases	BAC 0 mg/dL (%)	0 < BAC < 50 mg/dL (%)	BAC ≥ 50 mg/dL (%)
January–April	99	56 (56.6)	4 (4.0)	39 (39.4)
May–August	687	207 (30.1)	20 (2.9)	460 (67.0)
September–December	181	81 (44.8)	4 (2.2)	96 (53.0)
Total	967	344 (35.6)	28 (2.9)	595 (61.5)

*BAC* blood alcohol concentration

In comparison of BAC, VAC, and UAC, no cases that were both BAC-positive and VAC-negative appeared. Only four victims (0.4% of unintentional drownings) were BAC-positive and urine-negative. Such cases, all with BAC < 50 mg/dL (Table 3), raise the suspicion of PM alcohol production. These drownings occurred in March, April, June, and August^1^ (see Additional file 1) and the victims’ PM time of submersion ranged from less than 1 day to 50 days. Worth mentioning is that among cases with PM submersion time ≥ 7 days, of which more than half occurred during the warm season, of 52 cases, 50 showed no evidence of PM ethanol production.

**Table 3:** Unintentional drowning, 2000–2013: potential cases with PM ethanol production

Age (yrs), all male	BAC (mg/dL)	UAC (mg/dL)	VAC (mg/dL)	Month of death	Submersion time (days)	Activity, site	Witnesses
54	46	0	NA	April	0	falling, sea	NA
56	36	0	NA	June	3	falling, pond	no
48	25	0	NA	August	24	boating, lake	no
2	48	0	NA	March	50	falling, river	yes

*BAC* blood alcohol concentration, *UAC* urine alcohol concentration, *VAC* vitreous humour concentration, *NA* not available

Other 619 (99.4%) cases that were BAC-positive, alcohol was detectable also in urine or vitreous humour or in both, suggesting AM ingestion.

## Discussion

Alcohol is the most important single contributing factor in adult drowning (Driscoll et al. 2004; Lunetta et al. 2004; Peden et al. 2016; Pajunen et al. 2017). The present survey shows that 61.5% of the victims in Finland had BAC ≥ 50 mg/dL, a percentage consistent with findings of earlier studies performed in Finland (Lunetta et al. 2004; Pajunen et al. 2017).

PM endogenous alcohol production can, in principle, lead to overestimation of both the number of alcohol-positive drownings in large series and the actual BAC at time of death in particular cases. Some authors have suggested the use of cut-off times between death and sampling for alcohol analysis (ranging from 6 h to 72 h), in order to exclude cases with potential PM alcohol production (Schuman et al. 1978; Hoxie et al. 1988; Wintemute et al. 1990). Such cut-off times can, however, considerably reduce the percentage of actual drownings considered alcohol-positive. In the present study, use of a 24-h cut-off would exclude at least 20.5% of alcohol-positive drownings, a 48-h cut-off 12.4%, and a 72-h cut-off 11%.

Other drowning studies exclude low BAC values (< 50 mg/dL) to avoid any bias related to PM alcohol production (Ahlm et al. 2013; Peden et al. 2017). In Finland in 2000–2009, approximately 60% of alcohol-positive unintentional drowning victims had BAC ≥ 200 mg/dL (Pajunen et al. 2017), and in this survey, only 28 (2.9%) had BAC > 0 and < 50 mg/dL. Exclusion of cases with BAC < 50 mg/dL would therefore in Finland have had a less striking effect than had we used cut-off times. In countries reporting drowning to occur at lower BAC (Warner et al. 2000; Browne et al. 2003; Peden et al. 2017), exclusion of cases with low BAC may, however, have more tangible effects on the percentage of alcohol-positive drowning. Moreover, this approach would hamper studies on the effects on drowning of low BAC, with low BAC also being a potential risk factor for unintentional injuries (Ling et al. 2010; Amlung et al. 2014).

In the present survey, the higher percentage of drowning with BAC < 50 mg/dL during the summer months and in bodies with protracted PM submersion time may suggest a relationship between temperature- and time-dependent putrefaction and PM alcohol production. This hypothesis, nevertheless, was not demonstrated in our series. Indeed, several factors, including pre-drowning activities, exposure to water, patterns of alcohol consumption in aquatic settings, and circumstances of drowning (Lunetta et al. 2004; Bessereau et al. 2015), may well explain BAC differences across the seasons.

No studies have thus far addressed PM alcohol production in large series of drownings by comparing BAC, UAC, and VAC (Hadley and Smith 2003). PM ethanol production is usually presumed when BAC is positive and UAC and VAC are negative (Levine et al. 1993). Indeed, urine and vitreous humour are less prone to PM ethanol production than is blood, because these substrates do not contain—in a healthy individual—a significant amount of glucose, and the risk for micro-organism contamination is lower than in blood (FINE 1965; Kugelberg and Jones 2007; Belsey and Flanagan 2016).

Interestingly, the current study disclosed only four cases with potential PM ethanol production. These drownings represented 0.4% of overall unintentional drownings but surprisingly as much as 14.3% of drownings with BAC < 50 mg/dL, higher than the corresponding percentage (8%) in a US study including 381 forensic autopsies with BAC < 50 mg/dL (Levine et al. 1993).

Some caution is essential regarding the interpretation of these results. First, victims who were BAC-positive and UAC-negative may have ingested alcohol shortly before drowning (Jones 2006). The lack of measured VAC concentrations among these cases hampers verification of this hypothesis. Alcohol ingestion shortly before death may have occurred, for instance, in one unwitnessed cold-water drowning with PM submersion time < 24 h, in which the victim’s BAC was 46 mg/dL and he was UAC-negative. In vitro experiments show that PM endogenous ethanol production does not generally occur in temperatures < 4–5 °C, although it is still possible (Vuori et al. 1983) and takes place at the earliest after 24 h (Boumba et al. 2012; Boumba et al. 2013). Since vitreous humour was unavailable, the interpretation of this case remains a matter for speculation, however. Therefore, vitreous humour, when ever possible, should always be sampled at autopsy and tested for alcohol.

Second, we did not consider UAC/BAC ratios. UAC/BAC ratios < 1 may suggest drowning during the ethanol-absorptive phase (Jones 1992) or, alternatively, suggest PM ethanol production in urine or passive diffusion of ethanol from the gut to the urinary bladder (Kugelberg and Jones 2007). UAC/BAC ratios in drowning victims will be addressed in an ongoing study by our research group. Third, the presence of alcohol in urine and vitreous humour do not always indicate AM ingestion, because PM ethanol production may occur in these substrates, for instance in those victims with diabetes or in bodies with extensive injuries (Kugelberg and Jones 2007). As a further limitation, the present study allowed identification of drowning cases in which alcohol in victims’ blood may have been produced entirely PM; such data were of no value in evaluating overestimation of BAC, because of PM ethanol production in cases who had indulged in actual AM consumption.

In medico-legal cases involving potential criminal or civil litigation, a detailed case-by-case assessment of PM ethanol production remains crucial. It should include evaluation of the victim’s medical history and the circumstances leading to drowning, and also comparison of BAC, UAC, VAC, and analysis of putrefactive alcoholic indicators (e.g. n-propanol) and of ethanol metabolites (e.g. glucuronide), as well as including bacterial culture, molecular analysis, and fermentation test to identify micro-organism capable of producing PM ethanol (Vuori et al. 1983; Moriya and Hashimoto 2004; Ziavrou et al. 2005).

The present survey, however, suggests that, at least in Finland, PM endogenous alcohol production has a limited impact on epidemiological research on drowning and alcohol. Using too rigorous cut-off criteria, such as those previously recommended, may lead to significant underestimation of actual alcohol-positive drowning cases. This assumption should be verified by means of combined blood, urine and vitreous humour testing in other countries and settings, where higher ambient temperature and protracted PM submersion times may boost the putrefaction process and enhance PM alcohol production.

## Additional file

Additional file 1: Inland waters mean temperatures in Celsius degrees during years 2000, 2006 and 2013. (DOCX 15 kb)

## Abbreviations

AM: Ante-mortem
BAC: Blood alcohol concentration
PM: Post-mortem
UAC: Urine alcohol concentration
VAC: Vitreous humour alcohol concentration

## Footnotes

1 The surface temperatures of inland waters are measured during the open water season by Finnish Environment Institute. In Finland the inland waters typically freeze in November–December and the ice break-up in April–May. The peak temperatures are reached in July and August. The lowest mean, mean and highest mean temperatures (°C) of inland water in years 2000, 2006 and 2013 are presented in Additional file 1.

## References

[CR1] Ahlm K, Saveman BI, Bjornstig U (2013). Drowning deaths in Sweden with emphasis on the presence of alcohol and drugs - a retrospective study, 1992-2009. BMC Public Health.

[CR2] Amlung MT, Morris DH, McCarthy DM (2014). Effects of acute alcohol tolerance on perceptions of danger and willingness to drive after drinking. Psychopharmacology.

[CR3] Appenzeller BM, Schuman M, Wennig R (2008). Was a child poisoned by ethanol? Discrimination between ante-mortem consumption and post-mortem formation. Int J Legal Med.

[CR4] Belsey SL, Flanagan RJ (2016). Postmortem biochemistry: current applications. J Forensic Legal Med.

[CR5] Bessereau J, Fournier N, Mokhtari T, Brun PM, Desplantes A, Grassineau D (2015). Epidemiology of unintentional drowning in a metropolis of the French Mediterranean coast: a retrospective analysis (2000-2011). Int J Inj Control Saf Promot.

[CR6] Boumba VA, Economou V, Kourkoumelis N, Gousia P, Papadopoulou C, Vougiouklakis T (2012). Microbial ethanol production: experimental study and multivariate evaluation. Forensic Sci Int.

[CR7] Boumba VA, Kourkoumelis N, Gousia P, Economou V, Papadopoulou C, Vougiouklakis T (2013). Modeling microbial ethanol production by E. coli under aerobic/anaerobic conditions: applicability to real postmortem cases and to postmortem blood derived microbial cultures. Forensic Sci Int.

[CR8] Browne ML, Lewis-Michl EL, Stark AD (2003). Unintentional drownings among New York state residents, 1988-1994. Public Health Rep.

[CR9] Corry JE (1978). A review. Possible sources of ethanol ante- and post-mortem: its relationship to the biochemistry and microbiology of decomposition. J Appl Bacteriol.

[CR10] Driscoll TR, Harrison JE, Steenkamp M (2004). Alcohol and drowning in Australia. Inj Control Saf Promot.

[CR11] FINE J (1965). Glucose content of Normal urine. Br Med J.

[CR12] Gilliland MG, Bost RO (1993). Alcohol in decomposed bodies: postmortem synthesis and distribution. J Forensic Sci.

[CR13] Hadley JA, Smith GS (2003). Evidence for an early onset of endogenous alcohol production in bodies recovered from the water: implications for studying alcohol and drowning. Accid Anal Prev.

[CR14] Hoxie P, Cardosi K, Stearns M, Mengert P (1988). Alcohol in fatal recreational boating accidents.

[CR15] Jones AW (1992). Ethanol distribution ratios between urine and capillary blood in controlled experiments and in apprehended drinking drivers. J Forensic Sci.

[CR16] Jones AW (2006). Urine as a biological specimen for forensic analysis of alcohol and variability in the urine-to-blood relationship. Toxicol Rev.

[CR17] Kugelberg FC, Jones AW (2007). Interpreting results of ethanol analysis in postmortem specimens: a review of the literature. Forensic Sci Int.

[CR18] Levine B, Smith ML, Smialek JE, Caplan YH (1993). Interpretation of low postmortem concentrations of ethanol. J Forensic Sci.

[CR19] Ling J, Stephens R, Heffernan TM (2010). Cognitive and psychomotor performance during alcohol hangover. Curr Drug Abuse Rev.

[CR20] Lunetta P, Smith GS, Penttila A, Sajantila A (2004). Unintentional drowning in Finland 1970-2000: a population-based study. Int J Epidemiol.

[CR21] Moriya F, Hashimoto Y (2004). Postmortem production of ethanol and n-propanol in the brain of drowned persons. Am J Forensic Med Pathol.

[CR22] Pajunen T, Vuori E, Vincenzi FF, Lillsunde P, Smith G, Lunetta P. Unintentional drowning: role of medicinal drugs and alcohol. BMC Public Health. 2017;17(1):388. 10.1186/s12889-017-4306.8. 10.1186/s12889-017-4306-8 PMC5437510 28521790

[CR23] Peden AE, Franklin RC, Leggat PA (2016). The hidden tragedy of Rivers: a decade of unintentional fatal drowning in Australia. PLoS One.

[CR24] Peden AE, Franklin RC, Leggat PA (2017). Alcohol and its contributory role in fatal drowning in Australian rivers, 2002-2012. Accid Anal Prev.

[CR25] Schuman SH, Ollison CW, Wannamaker CC, Poston JH (1978). Which drownings are preventable? An in depth study of 62 deaths in Charleston County, South Carolina. J S C Med Assoc.

[CR26] Vuori E, Renkonen OV, Lindbohm R (1983). Validity of post mortem blood alcohol values. Lancet.

[CR27] Warner M, Smith GS, Langley JD (2000). Drowning and alcohol in New Zealand: what do the coroner's files tell us?. Aust N Z J Public Health.

[CR28] Wintemute GJ, Teret SP, Kraus JF, Wright M (1990). Alcohol and drowning: an analysis of contributing factors and a discussion of criteria for case selection. Accid Anal Prev.

[CR29] Ziavrou K, Boumba VA, Vougiouklakis TG (2005). Insights into the origin of postmortem ethanol. Int J Toxicol.

