# Supplementary material for: Epidemiology of alcohol-related unintentional drowning: is post-mortem ethanol production a real challenge?
Source: Inj Epidemiol. 2018 Nov 1;5:39. doi: 10.1186/s40621-018-0169-4 (PMC6211144; doi:10.1186/s40621-018-0169-4)
Supplement: Supplementary file 1 — Inland waters mean temperatures in Celsius degrees during years 2000, 2006 and 2013. (DOCX 15 kb) [file 40621_2018_169_MOESM1_ESM.docx]

Additional file 1

|  | **May** | **June** | **July** | **August** | **September** | **October** | **November** |
| --- | --- | --- | --- | --- | --- | --- | --- |
| **2000** | 9.2  (3.9-14.2) | 13.3 (3.7-18.1) | 18.4 (8.4-20.7) | 16.9 (10.9-19.7) | 11.9 (8.5-15.5) | 8.5 (6.2-10.1) | 3.8 (1.7-6.6) |
| **2006** | 9.4 (5.4-12.2) | 15.0 (6.7-18.3) | 18.7 (10.1-21.5) | 18.7 (12.3-21.4) | 13.5 (9.5-16.5) | 7.6 (3.6-11.0) | - |
| **2013** | 11.5 (6.0-18.4) | 17.5 (6.4-20.3) | 18.1 (10.5-20.4) | 17.8 (12.3-20.1) | 13.6 (10.1-16.6) | 6.1 (2.3-9.7) | 3.1 (0-5.7) |

Inland waters mean temperatures in Celsius degrees during years 2000, 2006 and 2013. Adapted from Finnish Environment Institute: syke.fi/avoindata
